# A life put on hold? Navigating fertility-related considerations after cancer in adolescence and young adulthood (AYA)

**DOI:** 10.1007/s00520-025-09832-9

**Published:** 2025-08-11

**Authors:** Taylor M. Dattilo, Leah Waterman, Larry L. Mullins, Vicky Lehmann

**Affiliations:** 1 Center for Pediatric Psychology, Oklahoma State University, Stillwater, OK 74078 USA; 2 Department of Medical Psychology, Amsterdam University Medical Center, University of Amsterdam, Amsterdam, 1105AZ The Netherlands; 3 Cancer Center Amsterdam (CCA), Amsterdam, 1105AZ The Netherlands

**Keywords:** Adolescence and young adulthood (AYA), Oncology, reproductive goals, Parenthood considerations, Fertility-related uncertainty, Fertility-related concerns, cancer survivorship

## Abstract

**Purpose:**

To examine fertility-related considerations of patients and survivors diagnosed with cancer during adolescence and young adulthood (AYA). Such fertility-related considerations include perceptions of putting reproductive goals “on hold” and subsequent effects on romantic relationships, parenthood considerations (i.e., arguments for/against having children), fertility-related uncertainty, and fertility-related concerns.

**Methods:**

An online cross-sectional survey was completed by of *N* = 190 patients/survivors of AYA cancer (*M*_age_ = 32.5 years, 87% female, diagnosed between ages 12–39 years; 65% were without biological children at study participation).

**Results:**

The majority of participants (72%) likely wanted to have children. Among them, 46% felt like their reproductive goals were put on hold due to cancer, causing negative emotions and negative effects on romantic relationships (e.g., pressure). Arguments against parenthood were predominantly *cancer-specific* (e.g., concerns/worries about health risks), but also *generic* (e.g., loss of freedom, sustainability). Arguments in favor of having children were almost all *generic* (e.g., meaning making). Fertility-related uncertainty caused negative emotions (e.g., sadness, insecurity, anxiety, feeling less human), and was correlated with various fertility-related concerns (*r* = .3–.7).

**Conclusion:**

Perceptions of putting reproductive goals “on hold” can affect well-being and romantic relationships, and fertility-related uncertainty may cause various negative emotions among young cancer patients/survivors. Arguments against parenthood evolve around cancer-specific concerns, which should be addressed by healthcare providers throughout survivorship. Providers are encouraged to provide patient-specific fertility-related education and psychological support if indicated.

**Supplementary Information:**

The online version contains supplementary material available at 10.1007/s00520-025-09832-9.

## Introduction

Undergoing cancer treatment during adolescence and young adulthood (AYA) can cause fertility problems or infertility due to exposure to gonadotoxic treatments [1–5]. Additionally, a cancer diagnosis throughout the AYA life phase may also disrupt the timing of young patients’ developmental milestones, such as establishing romantic partnerships or starting a family [6, 7]. Therefore, young cancer patients and survivors may regard possible parenthood differently, but systematic research is limited across this distinct yet diverse cancer population.

Fertility-related studies of patients with cancer during reproductive age often focus on specific diagnostic groups (e.g., testicular, gynecological, or breast cancer [8–10]) rather than considering AYAs with various types of cancer as a group with unique age-specific needs and a shared developmental life stage [11–13]. Research shows that young survivors often worry and express concerns about their fertility [14–23], struggle with romantic relationships when faced with possible infertility [7, 24–26], and they can have misperceptions or limited knowledge about their fertility [24, 27–30]. Such lack of knowledge may exacerbate uncertainty [28, 31–33], which may increase fertility-related concerns in turn. This is expected based on the general uncertainty literature, which highlights various unknowns in the medical setting [34]. Within the scope of this study, such uncertainties may pertain to unknown fertility status, possible decline in fertility potential over time, or unclear reproductive options [32, 33], which may all cause fertility-related concerns in turn. Yet, the connection between fertility-related uncertainties and concerns throughout cancer survivorship remains understudied and many of the above findings are based on smaller-scale qualitative studies [15, 18, 21, 24, 28, 32, 33].

At the same time, research has also shown that some survivors report reconsidering their life goals [18, 35] and finding joy in a life without children [36]. However, survivors’ parenthood considerations (i.e., arguments against *and* in favor of having children after cancer) remain understudied.

The current study included patients and survivors of AYA cancer to assess their fertility-related considerations, including their (1) perceptions of putting reproductive goals “on hold” and subsequent effects on romantic relationships, (2) parenthood considerations (i.e., arguments against/in favor of getting children), and (3) fertility-related uncertainty, associated emotions, and associations with fertility-related concerns. We combined structured and open-ended questions, allowing for quantitative and qualitative analyses, to gain novel insights into complex fertility-related considerations and associated emotional effects.

## Methods

### Study design

Data are part of the FROSA-study (FROSA = Fertility, ROmance, and Sex after cancer in young Adulthood), which recruited participants through online convenience sampling including social media posts via various Dutch and Belgian cancer patient support organizations, foundations, advertising on local news stations, and word of mouth via study participants. FROSA targeted Dutch-speaking survivors who had been diagnosed with cancer between ages 18–39 years, and who had completed treatment. Throughout recruitment, the study team was also approached by survivors diagnosed before age 18 and patients still undergoing treatment who also wanted to participate, which we did not oppose given their expressed importance of examined topics in this study.

The FROSA-survey was developed with a patient-centered approach and reviewed by experts in the field and several survivors who volunteered to provide feedback to the research team, which was incorporated accordingly. The FROSA-survey was anonymous, and participants provided written informed consent online before starting the survey. The medical ethics committee of the Amsterdam UMC approved this study as exempt from in-depth review (W21_282#21.309) and all procedures were in accordance with the Declaration of Helsinki for Medical Research involving Human Subjects.

### Materials

#### Reproductive goals and effects on romantic relationships

Participants were asked about their current reproductive goals, i.e., whether they wanted to have (additional) children (*yes*, *no*, *maybe*). Those who reported they might wanted to have children (i.e., answered *yes*/*maybe*) were asked whether their cancer diagnosis had put their reproductive goals on hold. This was specified with whether participants would have started to (try) having children earlier or around the time of their cancer diagnosis and directly assessed as such by a single item (*yes*/*no*). One additional open-ended item assessed how putting reproductive goals on hold influenced their dating/romantic relationships (see Appendix 1).

#### Parenthood considerations

Participants were briefed that any person might weigh arguments in favor or against possibly having children, and they were asked which aspects may play(ed) a role for them. Participants had the option to write as many arguments/reasons as they wanted under the prompts “arguments in favor” and “arguments against” having children (see Appendix 1).

#### Fertility-related uncertainty and emotional effects

One face-valid item assessed how uncertain participants felt about their fertility. Item responses ranged on a 5-point Likert-type scale from *not at all* (1) to *very much* (5)*.* Participants that endorsed at least *a little* uncertainty (i.e., scores ≥ 2) were also asked to complete the following sentence: “Uncertainties about my fertility make me feel _ _ _.”

#### Fertility-related concerns

Parts of the validated Reproductive Concerns After Cancer (RCAC) scale [37, 38] were used to assess fertility-related concerns. All items were translated into Dutch and double-checked with another research group that was working to validate a Dutch version of the RCAC in the Netherlands (including back-and-forth translations). Given overlap in some item-wording in the Dutch language (e.g., little difference in being worried vs. concerned) and to keep the FROSA-survey as short as possible, one item from each of the six RCAC-subscales was used (see Appendix 1) and will be reported accordingly: *fertility potential*, *partner disclosure*, *child health*, *personal health/relapse*, *acceptance of possible infertility*, and *achieving pregnancy* (where the male vs. female item version specified becoming pregnant vs. achieving a pregnancy). Three additional items were included and assessed whether participants were in a *hurry* to have children after treatment, whether they would feel less *feminine/masculine* if they could not have children, and (for those with knowledge of potential fertility problems) whether they find/found it more *difficult to find a partner*. Item response options were kept from the original RCAC and included a 5-point Likert scale ranging from *disagree completely* to *agree completely* (see Appendix 1).

### Statistical analyses

Data were analyzed using SPSS Statistics by IBM, version 28. Descriptive statistics were used to summarize participant responses. Differences in reproductive goals, fertility-related uncertainty, and concerns were examined by demographic and cancer-related factors using *χ*^2^-tests, *t*-tests, or one-way ANOVAs, depending on the used variables. Significance testing for *t*-tests included 95% confidence intervals yielded from 1000 bootstrapped resampling. Significance testing for continuous variables was also accompanied by calculating Hedge’s *g* effect sizes to estimate the magnitude of differences. Hedge’s *g* is interpreted like Cohen’s *d* (*g* ≥ 0.2 is small, ≥ 0.5 moderate, ≥ 0.8 large), but uses a correction to prevent overestimation.

All answers to open-ended questions were qualitatively analyzed by means of content analysis. Their salient content was independently double-coded by two coders (LW & VL) in a bottom-up manner (i.e., based on the apparent content). Codes were subsequently discussed and categorized into higher-order categories, which were also discussed among all authors and are reported accordingly.

## Results

### Participants

A total of *N* = 190 patients/survivors of AYA cancer completed the online survey. They were about 32.5 years of age (*SD* = 6.5, range 18–53) and the majority was female (87%, *n* = 165), partnered (74%, *n* = 140), and had no biological children at study participation (65%, *n* = 123). Participants had been diagnosed with cancer between ages 12–39 years, including *n* = 28 (14.7%) who also experienced relapses or second malignancies. Time since last diagnosis ranged from 0 to 24 years (*M* = 3.7, SD = 4.0) and most participants had completed active cancer treatment (93%, *n* = 176). Types of diagnoses included breast cancer (33%, *n* = 63), leukemia/lymphoma (30%, *n* = 56), gynecological cancers (15%, *n* = 28), and other types of cancer (22.7%, *n* = 43; including *n* = 11 with testicular cancer and *n* = 11 with sarcomas).

### Reproductive goals on hold

At the time of study, 45% (*n* = 85) of participants wished to have (more) biological children, 28% (*n* = 52) indicated “maybe,” and another 28% (*n* = 52) did not want to have children (of which *n* = 18 already had children). Of those participants who (yes/maybe) wished to have children, almost half (46%, *n* = 63/137) felt like their reproductive goals were put on hold due to cancer. Those participants were both older at study (*M* = 32.8 vs. 28.3; *t*(135) = 5.54, *p* < 0.001, *MΔ* = 4.6, 95% CI[3.1–6.0], *g* = 0.9) and older at diagnosis (*M* = 30.1 vs. 24.9; *t*(133) = 6.50, *p* < 0.001, *MΔ* = 5.2, 95% CI[3.6–6.6], *g* = 1.1). Men and women did not differ in their perceptions of being put on hold (*χ*^*2*^(1, *n* = 137) = 0.01, *p* = 0.924). The majority of participants who already had biological children still felt like their plans were put on hold (67%, *n* = 22/33), whereas the majority of participants without biological children did *not* feel like their plans were put on hold (61%, *n* = 63/104; *χ*^*2*^(1, *n* = 137) = 7.49, *p* = 0.006). Perceptions of putting reproductive goals on hold appeared different by type of diagnosis (*χ*^*2*^(3, *n* = 137) = 13.66, *p* = 0.003), such that most breast cancer patients/survivors felt being put on hold due to cancer (66%, *n* = 27/41), while most leukemia/lymphoma survivors did not (73%, *n* = 35/48).

The most common responses to how such stalled reproductive goals influenced their dating/relationships were (1) negative emotions, such as feelings of disappointment, sadness, anxiety, and feelings of inferiority. Participants also described added (2) pressure on romantic relationships, which included conflicts and sometimes led to break-ups. Other survivors reported experiencing (3) little or (4) no effects, for example due to not being busy with family planning (yet) or due to focusing on their own recovery/health. Positive effects, albeit a painful experience, included (5) better communication with romantic partners, feeling a sense of closeness, and a sense of living in the present (see an overview of all 5 identified categories in Table 1).

**Table 1:** Overview of how putting reproductive goals on hold influenced romantic relationships

	Reproductive goals being put on hold	n*
**(1)**	**Negative emotions: disappointment, sadness, anxiety, and feelings of inferiority**	16
	*E.g.: less worthy for a (potential) partner, guilt toward partner, difficult to adjust to new reality/adjust life goals; difficult when others have children*	
**(2)**	**Pressure on relationship**	7
	*Grew apart, biggest source of conflict, whole relationship feels ‘on hold’*	
	**(2a) Break-up/Divorce**	10
	*E.g.: situation magnified different attitudes toward reproductive goals, difficult to communicate; clarified that alternatives to biological parenthood are not an option*	
**(3)**	**Little effect**	13
	*E.g.: being happy that having children is possible, irrespective of timing; weren’t thinking about children yet*	
**(4)**	**No effect**	10
	*E.g.: recovery is more important*	
**(5)**	**Better communication/getting closer**	8
	*E.g.: although it’s painful, both are on the same page; partner also a source of comfort*	
	**Other**	7
	*E.g.: had kids right after treatment, freeing: less pressure about future, living in the present, will see what the future brings*	

*Number of times mentioned

*n*=63 participants indicated that their reproductive goals were put on hold due to cancer, of which almost all (*n*=62) provided written answers to an open-ended question about *how* having their reproductive goals being put on hold influenced their dating/relationships, *n*=47 provided answers specific to romantic relationships and are presented here

### Parenthood considerations (pros/cons of having children)

Participants’ arguments against having children were predominantly *cancer-specific*, which included concerns about their chances of relapse or early death and leaving children and/or a partner behind. Similarly, worries about whether they would be able/healthy enough to raise a child were indicated as well as worries about pregnancy complications (e.g., miscarriages), the complexities of possibly utilizing assisted reproductive technologies (ART), and the overall risks of pregnancies to their own health (e.g., possible implications of hormonal treatment). Moreover, genetic risks for cancer or other health risks for possible children were indicated as possible arguments against having children. Participants mentioned few *generic* reasons against having children, which included not wanting a big change in their lives or losing freedom, as well as sustainability (e.g., ecological footprint, climate change), financial insecurity, being appreciative of what they already had, increased age (either their own or their partner’s age), or the lack of desire to have children, among others (see Table 2).

**Table 2:** Overview of parenthood considerations (i.e., arguments against and in favor of having children)

**Arguments against having children**	n*
**Cancer-specific arguments**	
**Chance of relapse or early death; limited life expectancy**	54
* not wanting to leave a child (or partner with a child) behind; regrets if one would not be able to care for or see the child grow up*	
**Worries about whether one would be healthy enough to raise children**	44
* due to fatigue/low energy; raising a child would be too stressful/annoying; insecurities/doubts about being a bad parent (and what one would have to offer)*	
**Pregnancy too risky or complicated**	43
* assisted reproductive technologies (ART) would be too burdensome; worries about emotional burden of (repeated) miscarriages; previous negative experiences; worries about stopping hormonal treatments and inability to breast feed or give birth vaginally*	
**Genetic risks, mutation, or health of potential offspring**	27
**Insecurities about fertility**	7
* worries about being unable to get pregnant; decision to not try to get pregnant to save oneself from heartache; feelings of guilt towards partner*	
**Generic arguments**	
**Big change/responsibility or loss of freedom**	39
* less time (for work, relationship); less sleep*	
**Ecological footprint/climate, sustainability, overpopulation**	23
* there is too much misery in this world*	
**Finances/financial insecurity**	14
* Or needing a bigger house/more space*	
**Being happy with what you have** (e.g., being alone, with a partner, or with existing children)	15
**Not having a partner (or options) to have a child**	10
**Increased age** (own or partners’ age)	9
**No desire to have children**	6
**Having children considered as an egoistic choice**	6
**Other**	21
* (e.g., alternatives to biological parenthood are too complex; being unsure about reproductive goals, worries about possible regret, no support system; child should not become a caregiver later in life)*	

**Arguments in favor of having children**	***n***
**(Always wanted to) care for a child/become a parent, life goal**	88
* desire to create a legacy and recognize yourself in a child/see them grow up; being biologically wired to have children; wish to become a grandparent one day* **participants’ cancer treatment/fertility problems also reinforced their wish to have children; they believed they would be a good parent/teacher (after everything they have overcome); they also have the right to be a parent	
**Meaning-making, enriching, joy, happiness**	55
* appreciate and live life now/to the fullest; enjoying community, not being lonely*	
**Complete picture/wanting a family**	40
* including wanting to have a big family; adding a sibling (to existing children)*	
**Procreate with partner, expression of love with partner/having a family together with partner**	31
* Wanting to have a family with current partner; “allowing” partner to have children (of yours)*	
**(Unconditional) love**	23
Move on with life, having a good prognosis**	7
**Timing: otherwise, it might be too late**	7
**enough time has passed since cancer diagnosis	
**Other**	24
* (e.g., having the right partner; social pressure/not feeling left out (when everyone has children); having children as an insurance for later; not being alone later in life)*	

*Number of times mentioned; *n* = 116 participants provided at least one written response

**Indicates a cancer-specific reason amongst “generic” arguments

Arguments in favor of having children were predominantly *generic*, referring to life-long parenthood goals, meaning making and joy, expression of love, and having a family with their romantic partner. Interestingly, some participants mentioned to particularly wanting to move on with their lives, a good prognosis, or that enough time has passed since their cancer treatment to being ready to have children. Thereby, time and age may also play a crucial role (see Table 2).

### Fertility-related uncertainty and concerns

Uncertainty regarding their fertility ranged from 1 to 5 (*M* = 2.8, *SD* = 1.5). Women reported somewhat, but not statistically significant more uncertainty than men (*M* = 2.8 vs. 2.2; *t*(168) = 1.86, *p* = 0.065; *MΔ* = 0.62, 95% CI[− 0.04–1.27], *g* = 0.4). Participants without biological children experienced much greater uncertainty (*M* = 3.1 vs. 2.1; *t*(168) = 4.73, *p* < 0.001; *MΔ* = 1.06, 95% CI[0.62–1.50], *g* = 0.8). Yet, fertility-related uncertainty did not differ by type of diagnosis (*F*(3, 166) = 0.57, *p* = 0.633).

A total of 64% (*n* = 122) of all participants endorsed at least “a little” uncertainty about their fertility. Open-ended answers to how such uncertainty may make them feel revealed that it most commonly caused negative emotions, including feelings of sadness, insecurity, anxiety/doubts, feeling less feminine/masculine or inferior (to others that are able to procreate), restless while waiting for children, or powerless/hopeless (see Table 3 for an overview of all written responses).

**Table 3:** Open-ended responses to “Uncertainties about my fertility make me feel __ __ __”

	n*
**Sad, down, disappointed, less happy, broken**	41
**Insecure**	21
**Anxious, doubtful, scared, tense, stressed, worried**	21
**Less feminine, less masculine, mot manly, less myself**	14
**Less human, less worthy, inferior, incomplete**	8
**Indifferent, not different, OK**	7
**Restless, waiting**	6
**Powerless, helpless, hopeless**	6
**Different, abnormal**	5
**Lonely**	4
**Vulnerable**	4
**Old**	4
**Guilty**	3
**Less attractive**	2
**Rushed**	2
**Angry**	2
**Other**: jealous, never done, annoying, misunderstood, hopeful, regrettable, childless, resigned (each was mentioned once)	8

*Number of times mentioned; *n* = 113 participants provided responses, which often included several aspects

Fertility-related concerns were assessed on 10 domains (see descriptives in Table 4 and subgroup comparisons in Appendix 2). Several of these domains correlated with fertility-related uncertainty, including concerns about achieving a pregnancy (female, *r* = 0.67), concerns about fertility potential (*r* = 0.66), less acceptance of possible infertility (*r* =  − 0.50), partner disclosure (*r* = 0.41), difficulty finding a partner (*r* = 0.39), feeling less feminine (*r* = 0.35), hurry to have children (*r* = 0.34), and personal health/relapse (*r* =  − 0.17; Table 4).

**Table 4:** Descriptive statistics and bivariate correlations between various fertility-related uncertainty and various fertility-related concerns

		Fertility-related Uncertainty
	***M*** (SD), range	***r*** (***p***)
**Fertility-related uncertainty**	2.8 (1.5), 1–5	**-**
**Fertility-related concerns** regarding:		
Becoming pregnant (female)^+^	3.3 (1.4), 1–5	**0.670; *p* < 0.001**
Fertility potential^+^	3.4 (1.5), 1–5	**0.662; *p* < 0.001**
Acceptance of possible infertility^+^	3.3 (1.3), 1–5	** − 0.502; *p* < 0.001**
Partner disclosure of fertility status^+^	2.2 (1.5), 1–5	**0.405; *p* < 0.001**
Difficulty finding a partner	2.3 (1.3), 1–5	**0.388;* p* < 0.001**
Feeling less feminine (female)	3.0 (1.5), 1–5	**0.345; *p* < 0.001**
Hurry to have children	2.5 (1.5), 1–5	**0.344; *p* < 0.001**
Personal health/relapse^+^	3.0 (1.5), 1–5	** − 0.170; *p* = *0.0*26**
Feelings less masculine (male)	2.5 (1.3), 1–5	0.422; *p* = 0.050
Achieving pregnancy (male)^+^	2.8 (1.5), 1–5	0.319; *p* = 0.148
Child health^+^	2.8 (1.5), 1–5	− 0.015; *p* = 0.851

^+^RCAC item

## Discussion

The current study examined fertility-related considerations that patients and survivors of AYA cancer may face following their diagnosis. This included postponed reproductive goals, various cancer-specific and generic parenthood considerations, and rather moderate levels of fertility-related uncertainty, which were associated with negative emotions and various fertility-related concerns. These aspects together with their emotional impact on survivors and their romantic relationships deserve more attention in clinical care.

Many participants felt like their reproductive goals were put “on hold” due to cancer, particularly those who were older and those who had a desire to have (additional) children. These findings add to the previous literature underlining that cancer has the potential to disrupt socially valued milestones, such as family planning [6, 7] with possible negative effects on romantic relationships [7, 24–26]. Our open-ended questions added valuable insights, including that having to postpone reproductive goals caused various negative emotions among patients/survivors, such as feelings of inferiority and guilt for not being able to “provide” a child for a partner. Participants also reported cascading negative effects of postponed reproductive goals, which sometimes led to break-ups or divorce. Conversely, other participants voiced little or no effects, which appeared to be dependent on timing (e.g., early survivorship and prioritizing recovery; no active family planning yet). Thus, such negative effects may still occur in the future. Importantly, other participants felt they communicated better and became closer to their romantic partners and emphasized the importance of living in the present moment. Breast cancer patients/survivors in particular believed that their reproductive goals were put on hold more often than survivors of other types of cancers (i.e., leukemia/lymphoma). This is likely caused by adjuvant hormone therapy, which is typically given after breast cancer and limits women’s ability to have a child for an extended period of time, leaving them less likely to have children [39].

Various arguments for/against having children reiterated fertility-related concerns, which are partly also captured in the RCAC and reported in previous research [22, 37, 38]. These considerations serve as possible arguments against having children (e.g., worries about fertility potential, own health, and an offspring’s health). However, our analyses also extend previous research by highlighting additional worries, such as anxieties about repeated miscarriages, the possible physical burden of hormones and ART utilization, and fear of leaving a romantic partner or child behind. It is important to support survivors and patients in navigating fertility-related decisions by addressing these concerns and potential misperceptions (e.g., about ART, prognosis, risks for potential offspring) to help alleviate potential undue anxiety or burden. Moreover, generic arguments unrelated to participants’ cancer experiences also play an important role (e.g., loss of freedom, sustainability, financial aspects). As such, parenthood considerations are not exclusively tied to survivors’ previous disease, which should also be addressed in clinical care. Moreover, as the timing of a cancer diagnosis during peak reproductive years likely contributes to feeling pressure to have children sooner and may increase worries about survivors’ advancing age and fertility potential, these aspects of fertility should also be discussed with survivors and patients.

Arguments *in favor* of having children were predominantly of generic nature (i.e., unrelated to cancer), such as love and meaning making. These responses are generalizable to the viewpoints of any individual (with or without cancer), which also illustrates how cancer-specific concerns may be one piece in a more complex choice of reproduction and family building. This is important to be acknowledged with AYA cancer patients and survivors in clinical care, which also serves to normalize some of their experiences and fertility-related concerns.

Fertility-related concerns have been studied more often recently [14–23, 32]. However, we propose that fertility-related uncertainty may play a crucial role even before specific concerns arise, as patients and survivors are often faced with various unknowns and ambiguities regarding their fertility. As indicated in previous research, fertility-related uncertainty [28, 31–33] is sometimes driven by insufficient knowledge, which likely exacerbates fertility-related concerns. We extend these qualitative findings by showing moderate levels of fertility-related uncertainty (i.e., score 2.8 on a scale from 1 to 5) and by systematically assessing emotional implications of uncertainty. For example, participants described various negative emotions, including feelings of sadness, disappointment, insecurity, anxiety, or feeling less human. Thus, uncertainties should be systematically screened for and addressed in clinical care by providing psychoeducation and counseling to mitigate these subsequent negative emotions.

Notably, previous research has also found that increased uncertainty is associated with an increased interest in fertility testing in cancer survivorship [40, 41], likely in an effort to alleviate ambiguity. However, it is also possible that fertility testing produces inconclusive results (particularly for women), which often *exacerbates* worries and anxiety associated with fertility-related uncertainty [41]. Moreover, female survivors are often counseled about their shortened reproductive time window as they can be at risk for premature ovarian insufficiency (POI), which may increase the pressure to have children sooner [42], further contributing to increased fertility-related uncertainty. Uncertainty was correlated with various fertility-related concerns in this study, including fertility potential, partner disclosure (of fertility status), less acceptance of possible infertility, personal health/relapse, concerns about becoming pregnant (in women), hurry to have children, feeling less feminine, and difficulty finding a partner. Although we were not able to establish directionality of these relationships due to the cross-sectional nature of the study, our results demonstrate that uncertainties and concerns could reinforce each other. Thus, discussing one may improve the other, whereas discussing both issues in conjunction may be particularly helpful.

Standards of practice currently call for clinicians to provide better information regarding fertility after cancer, furthering the need for more counseling and perhaps training for healthcare professionals. However, clinicians often report multiple barriers to initiating these important reproductive health conversations with AYAs [43, 44]. To address these barriers, research examining evidence-based training strategies for providers has found that using scripts to initiate fertility-related conversations has been efficacious in helping providers feel more competent and prepared, while also prioritizing an individualized, tailored, multidisciplinary approach to care [45]. It may also be helpful to administer self-report measures before initiating conversations for additional guidance for providers. Based on this study, we also want to highlight that considering patients’ and survivors’ emotional states and particularly their feelings of inferiority is crucial in the face of parenthood considerations and effects on romantic relationships. Future research should continue to explore training programs for clinicians to feel comfortable and competent in facilitating reproductive health conversations and referrals to psychosocial care.

## Limitations

Our cross-sectional design limits our ability to infer causal relationships, but item-wording clearly instructed participants to answer questions in regard to having had cancer and effects on current reproductive goals, parenthood considerations, uncertainty, and concerns. Our sample size was sufficient to have adequately powered analyses, but larger subgroups (e.g., male vs. female, different types of diagnoses) may allow for more detailed analyses in the future. Although our sample size could limit the generalizability of our findings, the bootstrapping method used for *t*-tests reduced risks for type I error along with our Bonferroni correction for multiple testing of various fertility-related concerns (Appendix 2). The generalizability of our findings may also be limited by our Dutch-speaking sample and limited background information. Future research should also collect additional information about sociodemographic and cultural characteristics (e.g., race, sexual orientation) and medical variables (e.g., specific types of treatment) to better assess fertility-related considerations in survivorship. Additionally, the study design of online convenience sampling did not allow for response rate calculations, and likely caused selection bias, as people interested in our examined topics self-selected into participating.

## Conclusions

The current study examined fertility-related considerations including delayed reproductive goals and subsequent effects on romantic relationships and survivors’ emotional states, parenthood considerations, fertility-related uncertainty, and fertility-related concerns during and after AYA cancer. These patients and survivors share a developmental period, where reproductive goals are often put on hold with predominantly negative, but also some positive implications for romantic relationships. Fertility-related uncertainty is relatively common, which may easily be addressed in clinical care, especially as it relates to various fertility-related concerns as demonstrated in this study. Overall, we urge healthcare providers to consider multidisciplinary approaches to educate and counsel AYAs about fertility and family planning throughout cancer survivorship, where providers emphasize tailored and person-specific reproductive health conversations, which not only address physical aspects of (in)fertility, but also emotional states and any fertility-related question. This approach should also include fertility assessments, if needed, and support in coping with possible test outcomes (e.g., counseling for those with confirmed infertility about their loss of possible parenthood; continued surveillance for those with unclear test results). Patients and survivors of AYA cancer should also be referred to psychosocial providers or peer support options if needed.

## Supplementary Information

Below is the link to the electronic supplementary material.

Supplementary file 1 (PDF 224 KB)

## Data Availability

The de-identified data underlying and analyzed in the current study are available from the corresponding author on reasonable request.
