# Supplementary material for: A life put on hold? Navigating fertility-related considerations after cancer in adolescence and young adulthood (AYA)
Source: Support Care Cancer. 2025 Aug 11;33(9):779. doi: 10.1007/s00520-025-09832-9 (PMC12339621; doi:10.1007/s00520-025-09832-9)
Supplement: Supplementary file 1 — (PDF 224 KB) [file 520_2025_9832_MOESM1_ESM.pdf]

Appendix to the manuscript:

A Life Put on Hold? - Navigating Fertility-Related Considerations after Cancer in Adolescence and Young Adulthood (AYA)

Taylor M. Dattilo, Leah Waterman, Larry L. Mullins, & Vicky Lehmann

Contact:
Vicky Lehmann
Amsterdam UMC
email: v.lehmann@amsterdamumc.nl

FROSA

Overview:

Appendix-1: English translation of items used in the current study (as presented to participants) **page 2**

Appendix-2: Subgroup comparisons of fertility-related concerns **page 4**

Appendix-1:

English translation of items used in FROSA = Fertility, ROmance, and Sex in Young Adulthood

Note that aspects shaded in grey indicate automatic filters (i.e., questions were only shown to specific participants based on preceding answers)

Do you wish to have (additional) biological children?

- ☐ Yes, I/my partner are currently trying to get pregnant
- ☐ Yes, in the near future
- ☐ Yes, in the future/ long-term
- ☐ Maybe/ I don't really know yet
- ☐ No

If yes/maybe:

Did you put your wish to have children 'on hold' due to your cancer diagnosis?
In other words: Would you have had children earlier or right around the time of your diagnosis?

- ☐ Yes
- ☐ No

If yes:

How and to what extent did "being on hold" affect your dating/ romantic relationships?

…….

…….

Whether or not to have children influences the life of every human being to a great extent, and we are curious:

Which aspects play or played a role for you when it comes to decisions about whether or not to have children?
[enter as many arguments as you want]

Arguments **in favor**:	Arguments **against**:
……………………………………..	……………………………………..
………………………………………	………………………………………
………………………………………	………………………………………

What do you think about the statements below?

	Disagree completely	*Disagree somewhat*	*Neither agree nor disagree*	*Agree somewhat*	*Agree completely*
1. I'm afraid I won't be able to have any (more) children					
2. I am worried about passing on a genetic risk for cancer to my children					
3. I worry about telling my (potential) partner that I may be unable to have children					
4. I will be happy/satisfied with life whether or not I have (more) children					
5. Having (more) children would make me more anxious about getting cancer again					
6.<br>If female: It is stressful to think about trying to get pregnant<br>If male: It is stressful to think about trying to achieve a pregnancy					
7. I am in a greater hurry to start having a family, because I had cancer					
8.<br>If female: If I could not have biological children, I would feel less feminine<br>If male: If I could not have biological children, I would feel less masculine					
9.<br>If infertile/ having fertility problems:<br>It is or was more difficult for me to find a partner, because I (may) be unable to have biological children					

How uncertain do you feel about your own fertility?

- ☐ Not at all
- ☐ A little
- ☐ Somewhat
- ☐ A lot
- ☐ Very much

If 'a little' or higher:

Please complete the following sentence:
Uncertainties about my fertility make me feel: _ _ _ _ _ _ _ _ _ _ _ _ _ _ _

Appendix-2: Summary of subgroup comparisons of fertility-related concerns

Due to multiple comparisons of the 10 fertility-related concerns items, we used a Bonferroni correction to adjust the *p*-level to p=.005.

- Male and female participants differed on one fertility-related concern item, such that women reported feeling a greater hurry to start a family (M=2.6 vs. 1.8; $t(38.63)=3.29$, p=.002, $M\Delta$=0.84, 95% CI [3.2 - 1.36], g=0.6).

- Another item significantly differed by type of diagnosis: concerns about partner disclosure were significantly higher in participants with gynecological cancers (M=3.0) compared to all other diagnostic groups (*M*s<2.3, ($F(3, 185)=4.63$, p=.004; *g*s=0.4 - 0.7).

- The majority of participants who had biological children were less concerned about their fertility potential (M=2.9 vs. 3.7; $t(115.63)=3.39$, p=<.001, $M\Delta$=0.78, 95% CI [0.32 - 1.42], g=0.5), less concerned about partner disclosure (M=2.5 vs. 1.6; $t(178.30)=4.67$, p=<.001, $M\Delta$=0.89, 95% CI [0.51 - 1.27], g=0.6), less concerned about personal health/relapse (M=3.5 vs. 2.8; $t(187)=-3.32$, p=.001, $M\Delta$=-0.75, 95% CI [-1.19 - (-0.30)], g=0.5), and were more accepting of possible infertility (M=3.7 vs. 3.1; $t(187)=-3.24$, p=.001, $M\Delta$=-0.64, 95% CI [-1.04 - (-0.25)], g=0.5).
